# Aktina Vision: Full-parallax three-dimensional display with 100 million light rays

**DOI:** 10.1038/s41598-019-54243-6

**Published:** 2019-11-27

**Authors:** Hayato Watanabe, Naoto Okaichi, Takuya Omura, Masanori Kano, Hisayuki Sasaki, Masahiro Kawakita

**Affiliations:** Science & Technology Research Laboratories, NHK (Japan Broadcasting Corporation), Tokyo, 157-8510 Japan

**Keywords:** Displays, Optical techniques

## Abstract

Natural three-dimensional (3D) images, perceived as real objects in front of the viewer, can be displayed by faithfully reproducing light ray information. However, 3D images with sufficient characteristics for practical use cannot be displayed using conventional technologies because highly accurate reproduction of numerous light rays is required. We propose a novel full-parallax light field 3D display method named ‘Aktina Vision’, which includes a special top-hat diffusing screen with a narrow diffusion angle and an optical system for reproducing high-density light rays. Our prototype system reproduces over 100,000,000 light rays at angle intervals of less than 1° and optimally diffuses light rays with the top-hat diffusing screen. Thus, for the first time, light field 3D image reproduction with a maximum spatial resolution of approximately 330,000 pixels, which is near standard-definition television resolution and three times that of conventional light field display using a lens array, is achieved.

## Introduction

Light field displays have attracted attention as a three-dimensional (3D) display method that does not require glasses. In this method, the colour, intensity, and direction of light rays from objects are reproduced geometric-optically^1^. When observing a displayed light field image, a natural 3D image can be perceived as if there are real objects in front of the viewer because the main stereoscopic visual functions of human eyes such as binocular parallax, motion parallax, convergence, and accommodation are satisfied. Unlike holography^2–5^, which is based on wave-front reproduction, light field displays are based on incoherent light-ray reproduction and do not require a coherent laser beam for recording and reproducing optical images. They also do not require the use of a high resolution spatial light modulator with a pixel size close to the wavelength of visible light. Because of these advantages, light field displays are expected to find practical application in various fields such as TV broadcasting, medicine, education, gaming, 3D-CAD, and advertising.

Light field display methods can be broadly categorized into those that generate only a horizontal parallax and those that generate both horizontal and vertical parallaxes. As regards the former, display methods based on multi-view projection are being studied, where multi-view images are projected onto an anisotropic diffusing screen with different diffusion characteristics in the horizontal and vertical directions^6–14^. With the diffusing screen, the multi-view images are continuously displayed without gaps between viewpoints by diffusing the light rays slightly in the horizontal direction and widely in the vertical direction. Consequently, a horizontal parallax is generated, and the same 3D images can also be observed when moving in the vertical direction. Not only rear-projection-type display systems^6–10^ but also front-projection-type display systems using retro-reflective materials^11,12^ and 360° 3D display systems using cylindrical diffusing screen^13,14^ have been studied. High-resolution 3D images can be displayed because the amount of image information is increased by adding display devices. However, there are some issues in the image quality. First, 3D images cannot be perceived when viewers lean their heads because a vertical parallax is not generated. Second, distortions occur in the displayed images when observed at a different distance other than the designed viewing distance because the light rays are widely diffused in the vertical direction and optical images are not formed in the space. These two issues are not solved even when the head tracking technique^11,13^ is used. Finally, a holographic film with the Gaussian diffusion characteristics is generally used as the diffusing screen; thus, the spatial resolution characteristics of the 3D images at deep depths are degraded owing to a relatively large amount of crosstalk between the reproduced light rays.

Integral imaging methods based on integral photography^15^, which was invented by Lippmann in 1908, are mainly used for generating both horizontal and vertical parallaxes. In this approach, a lens array consisting of many small elemental lenses is placed in front of the display device. When elemental images are displayed on the device, light rays emitted from each pixel of the elemental images are incident on the corresponding elemental lenses and are refracted in prescribed directions. These light rays form an optical image in the space. Using this feature, a full parallax 3D image according to a viewing position can be obtained. Although, in principle, the pseudoscopic problem arises in the capture and display process, it can be avoided by using methods such as an electronic concave-convex converter^16^. However, an increase in the number of lenses and micronisation of both the lens and pixel size of the elemental images are needed to enhance the 3D image resolution because the resolution becomes less than the number of lenses constituting the lens array. Further, crosstalk between light rays, as described above, occurs when micronising the lens size to sub-millimetre sizes because diffraction effects significantly degrade the rectilinearity of the reproduced light rays. Crosstalk between light rays resulted in the degradation of the spatial resolution characteristics of 3D images at deep depths^17,18^. Various integral imaging methods using a high-definition display device^19^, multiple display devices^20–25^, and a time-division multiplexing technique^26–28^ for enhancing the display characteristics of 3D images have been proposed. However, the maximum resolution of the 3D images using these methods is approximately 100,000 pixels^19,23,24^, and a higher resolution has not yet been realised.

Considering the issues with these two types of display technologies, to further enhance the practicability of light field displays, a novel 3D display method, which is capable of generating both horizontal and vertical parallaxes and realising high display characteristics while reducing crosstalk between light rays, is needed. As a technology that can potentially solve these issues, a full-parallax 3D display method which uses a top-hat diffusing screen consisting of two orthogonal lenticular lenses and index-matching fluid was proposed^29^. However, because this diffusing screen has the two-layer structure, which probably degrades the image quality of the displayed 3D images because of defocusing, multiple reflections, and stray light in the screen. In addition, the display results using the screen with a diffusion angle of approximately 4.7° were reported; however, naturally viewable light field 3D images require high-density light rays with an angle interval less than 1°^10^.

**Figure 1:**
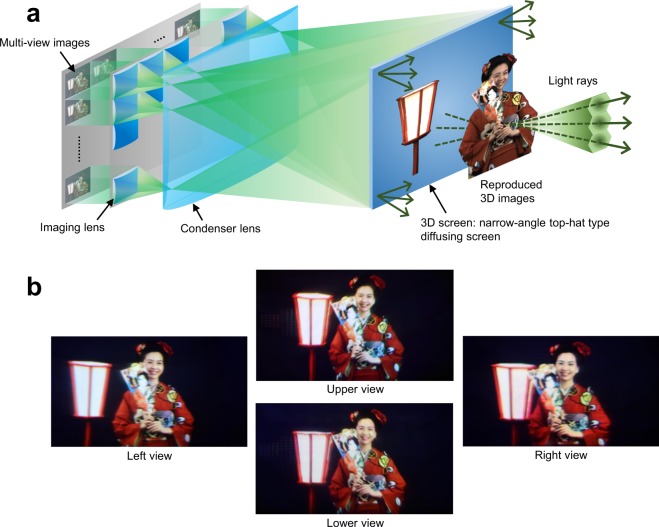
Aktina Vision with top-hat type diffusing screen. (**a**), Basic principle of Aktina Vision. An optical image of objects is reconstructed by projecting multi-view images with horizontal and vertical parallaxes at narrow angle interval onto the 3D screen and optimally diffusing light rays with the 3D screen. (**b**), Displayed 3D images from different viewpoints. Horizontal and vertical motion parallaxes are confirmed by the change in the positional relation such as between the woman, battledore, and lantern. Photo credits: Masanori Kano and Hayato Watanabe.

In this paper, we propose a full-parallax light field display method, named ‘Aktina Vision’, which consists of a 3D screen using a special diffusing screen with isotropic narrow diffusion characteristics and a display optical system for projecting high-density light rays. Using this method, light rays of 3D images are reconstructed from high-density multi-view images, as shown in Fig. 1a. The multi-view images, which have horizontal and vertical parallaxes and are arrayed in the prescribed plane, are projected onto the 3D screen from various angles in a superposed manner by the display optical system, which includes the imaging lens and condenser lens. The specially designed single-layer 3D screen has isotropic top-hat diffusion characteristics and a narrow diffusion angle of approximately 1° according to the projection angle interval of the light rays to slightly widen the discrete light rays. As a result, high-density light rays with a continuous luminance distribution are reproduced while reducing the crosstalk amounts between light rays.

In a prototype display system constructed based on Aktina Vision, over 100,000,000 light rays were reproduced using image information equivalent to 14 times that in a 4K resolution (3840 × 2160 pixels) image. Multi-view images with 768 × 432 pixels and 350 viewpoints were projected onto a 3D screen at an angle interval less than 1.0° in a superposed manner. In the fabrication of the 3D screen, we formed micro-lens shapes on the surface and controlled the diffusion angle by optimising the difference in refractive index between the lenses and their fillings. Light rays through the 3D screen are optimally diffused, and unlike conventional display systems based on multi-view projection^6–14^, the optical 3D images are formed in front of and behind the 3D screen by reproducing numerous high-density light rays.

Figure 1b shows a 3D image displayed using the prototype system, which is generated from multi-view images of real objects captured by the camera array system. A maximum spatial resolution of approximately 330,000 pixels, which is three times that of conventional lens array display methods, was realised for the first time. In addition, both horizontal and vertical parallaxes within a viewing angle of 35.1° in the horizontal direction and 4.7° in the vertical direction and moving 3D image reproduction with a frame rate of 60 fps were achieved.

## Results

### Design and fabrication of 3D screen

In light field display methods, it is empirically known that 3D images with a favourable motion parallax can be obtained by setting angle interval of light rays to less than 1°^10^. Light field displays use collimated light rays with high rectilinearity; however, in the case of the interval of approximately 1°, gaps occur between light rays such that their reproduction becomes discrete. Therefore, by widening the light rays at a necessary minimum, the luminance distribution of the 3D images when observing the light field must be uniform. However, if the widening angle is too large, the spatial resolution characteristics of the 3D images at deep depths becomes low because the crosstalk amount between adjacent light rays increases. For these reasons, by setting diffusion characteristics to top-hat shape rather than the general Gaussian shape, the spatial resolution characteristics can be enhanced while eliminating gaps between light rays and reducing the crosstalk amount.

**Figure 2:**
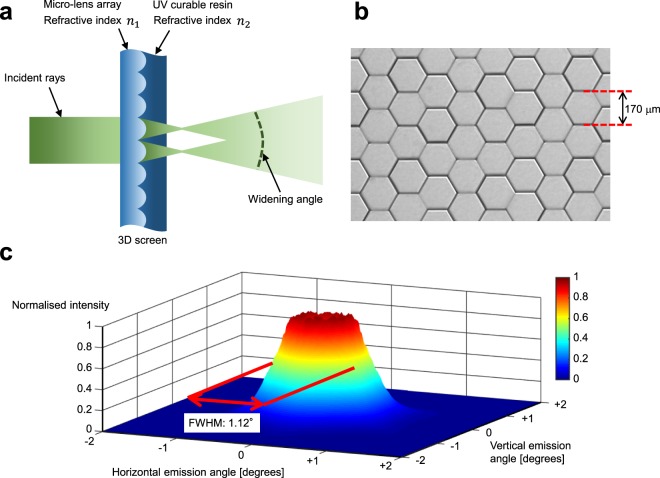
3D screen. (**a**), Configuration of the 3D screen. The fine lens structure, which is smaller than the pixel size of the projected image, is fabricated in a two-dimensional array shape with a UV-curable resin. Next, a UV-curable resin made of another material is used to fill in the surface and cured. By optimally selecting the UV-curable resin materials to adjust the difference in their refractive indices, the widening angle is controlled. (**b**), Enlarged image of the surface of the 3D screen. Hexagonal lenses with a size of 170 μm were formed in a delta arrangement. (**c**), Measurement results of the diffusion characteristics of the 3D screen. The diffusion characteristics resemble a top-hat shape with a narrow diffusion angle and a FWHM of approximately 1.0°. Photo credit: Hayato Watanabe.

To realise the diffusing screen with the narrow diffusion angle of approximately 1.0° and top-hat diffusion characteristics, we developed a special 3D screen whose surface is finely fabricated in micro-lens shapes. The configuration of the screen is shown in Fig. 2a. The lens structure, with a fine lens size smaller than the pixel size of the multi-view images projected onto the screen (under 500 μm), was fabricated in a two-dimensional array shape. The shape of one lens was hexagonal so that the diffusion angles were almost uniform in all directions and non-lens part was not formed. Further, the lens size was set to be 170 μm, such that the resolution of the displayed images is not reduced while restricting the generation of diffracted light rays with large diffraction angles. When a parallel light ray is incident on the 3D screen, widening of the light occurs according to the numerical aperture of the lens. Control of the light widening angle must be adjusted and optimised according to the angle interval between light rays in the display optical system. In the fabrication of the 3D screen, first, we manufacture a metal mould of hexagonal lens shape by an etching process; the metal mould is filled with a UV-curable resin and the lenses are formed by curing. Next, the formed resin of the lens array is filled with UV-curable resin made of another material with a different refractive index and is cured (Fig. 2b). The widening angle *θ* of the screen is given as follows:

1 $$\theta =2{\tan }^{-1}(\frac{r{\rm{\Delta }}n}{2R}),{\rm{\Delta }}n={n}_{1}-{n}_{2},$$

where *n*_1_ and *n*_2_ are the refractive indices of the lens and filling materials, respectively; *r* is the lens diameter; and *R* is the curvature radius of the lens. Finally, the widening angle of the light is controlled to the optimal value according to the display system by selecting curable resin materials for the lenses and their fillings to adjust the difference in refractive index Δ*n*. In our prototype system, we set the refractive indices of the lenses *n*_1_ and their fillings *n*_2_ to 1.60 and 1.50, respectively, the screen size to 488 mm × 274 mm, and lens size to 170 μm (Fig. 2b). Figure 2c shows the measurement results of the diffusion characteristics when white parallel light was incident on the screen. Diffusion characteristics close to a top-hat shape with a narrow diffusion angle and a full width at half maximum (FWHM) of approximately 1.0° were realised. The 3D screen widens light rays not by the diffraction effects but because of the refraction of the lenses. Therefore, the chromatic dispersion is low compared with holographic optical elements, and speckle noise is not observed.

### Development of display system based on Aktina Vision

**Figure 3:**
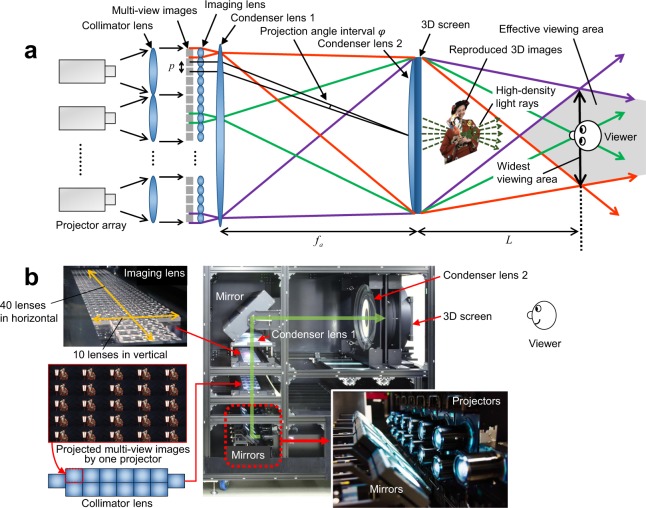
Prototype display system. (**a**), Configuration of the prototype display system. Multi-view images are incident on the imaging lens as parallel light and projected onto the 3D screen in a superposed manner at different angles while being enlarged. (**b**), Photograph of the prototype display system. By projecting multi-view images consisting of 350 viewpoints with 14 4K projectors, an optical 3D image is reconstructed by over 100,000,000 light rays. Photo credits: Masanori Kano and Hayato Watanabe.

Figure 3a shows the top view of the display optical system. In this method, image information consisting of a large number of pixels is necessary to reproduce many light rays and to reconstruct an ideal optical image. This is because each pixel of multi-view image corresponds to one light ray. Therefore, in the development of the prototype system, we reproduced numerous light rays by implementing multiple ultra-high definition (UHD) projectors. As shown in Fig. 3a, multiple projectors are placed in parallel, and each projector directly projects multi-view images consisting of 25 viewpoints (5 horizontal and 5 vertical). All multi-view images are projected without gaps by placing the projectors close to each other. Each multi-view image is enlarged and projected onto the 3D screen by the imaging lens and condenser lens. In Fig. 3a, the projection angle interval *φ* of the multi-view images is given by the following equation:

2 $$\phi \approx \frac{{p}_{k}}{{f}_{a}},$$

where *f*_*a*_ is the focal length of condenser lens 1 and *p*_*k*_(*k* = *h*,*v*) is the lens pitch of the imaging lens in the horizontal or vertical direction shown in Fig. 3a. Light rays with a continuous luminance distribution are reproduced by making the diffusion angle of the 3D screen and projection angle interval *φ* equal.

As shown in Fig. 3a, the reproduced light rays are condensed at the predetermined widest effective viewing area to efficiently use image information. By using this configuration, the density of the light rays was increased in the effective viewing area. The distance from the 3D screen to the widest effective viewing area *L* is given by

3 $$L=\frac{{f}_{a}\cdot {f}_{b}}{{f}_{a}-{f}_{b}}$$

where *f*_*b*_ is the focal length of condenser lens 2 and *f*_*b*_ is set to *f*_*b*_ ≤ *f*_*a*_. The longer the focal length *f*_*b*_, the longer the distance *L*. When *f*_*a*_ is equivalent to *f*_*b*_, parallel light rays are reproduced, which are geometric-optically the same as light rays reproduced with an ordinary integral imaging method. From an arbitrary point on the 3D screen, light rays were emitted in both horizontal and vertical directions at narrow angle intervals, represented by Eq. (2). Consequently, high-density light rays were emitted from the entire screen, and they formed an optical 3D image in front of and behind the 3D screen, unlike the conventional display methods based on multi-view projection^6–14^. Because of this feature, a full-parallax 3D image can be observed by the viewer from any position in the effective viewing area.

Figure 3b show the prototype system. We adopted a configuration in which light rays are returned by mirrors placed at two positions. Dedicated compact projectors with full 4K resolution were developed as display devices, and 14 such projectors were placed in parallel. One projector displayed multi-view images consisting of 25 viewpoints, 5 horizontal and 5 vertical, with a resolution of 768 × 432 pixels. By using 14 projectors to project multi-view images consisting of a total of 350 viewpoints onto the 3D screen at angle intervals of less than 0.93°, high-density light fields were reconstructed with over 100,000,000 light rays. The horizontal viewing angle was enlarged by projecting 40 multi-view images in the horizontal direction.

### Evaluating reproduced 3D images

**Figure 4:**
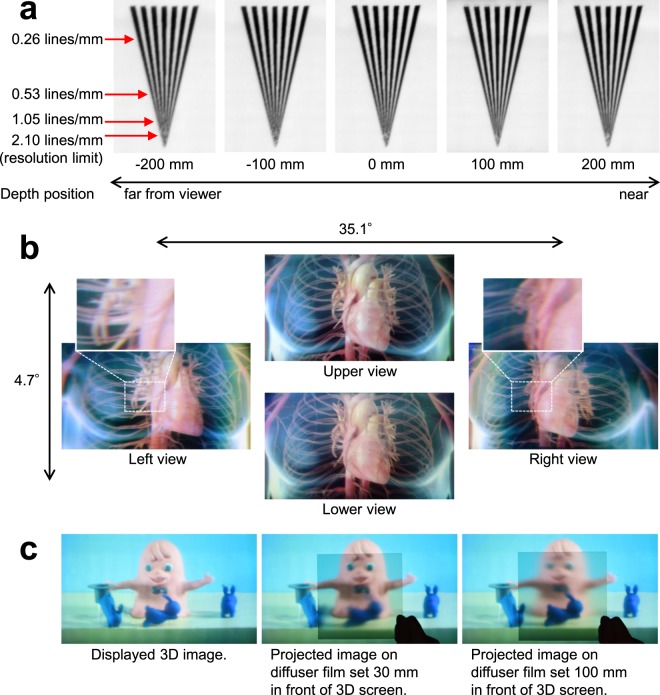
Displayed 3D images. (**a**), Displayed 3D images of a resolution chart. A resolution limit of 2.10 lines/mm, where folding distortion occurred, could be displayed. Even in the depth range of ±200 mm, resolution characteristics did not decrease significantly. This result show that viewers can observe 3D images with wide depth ranges. (**b**), 3D image of CG observed from different viewpoints. Motion parallax was confirmed by the change in the positional relation between the heart and blood vessels. The viewing angle is 35.1° in the horizontal direction and 4.7° in the vertical direction, and simultaneous viewing by multiple viewers is possible. (**c**), Projected images of reproduced light rays on the diffuser film. When the diffuser film was set at 30 mm and 100 mm in front of the 3D screen, the pink mascot character and blue rabbit, respectively, were projected clearly. This result shows that an optical image of objects can be reconstructed with Aktina Vision. Photo credits: Hayato Watanabe.

To verify the spatial resolution characteristics of the 3D images perceived by the viewer, we displayed a wedge chart and captured the display screen directly using a digital camera placed at a position of 1500 mm, which is the distance from the 3D screen to the widest effective viewing area *L* of the prototype system (Fig. 4a). The display position of the wedge chart was five steps, from 200 mm in the rear direction to 200 mm in the front direction with reference to the position of the 3D screen. The modulation factor of the wedge chart was reduced according to the increase in the spatial frequency of the pattern, and folding distortion occurred when 2.10 lines/mm, which is the upper limit of resolution, was exceeded. The cause of the modulation factor degradation is thought to be the aberration of lenses constituting the display optical system and the limit of condensing performance of the Fresnel lens used as condenser lens 1. In contrast, the spatial resolution characteristics of the captured 3D image did not significantly depend on the depth position of the 3D image. This result shows that the viewers can perceive 3D images with wide depth ranges. In Aktina Vision, multi-view images are condensed at the widest effective viewing area as shown in Fig. 4a. When observing the display screen from this area, the spatial resolution of 3D images perceived by the viewer is the same as that of multi-view images^30^. In the proposed system, the depth position of the 3D image does not affect the resolution because the crosstalk amount between multi-view images is minimized by the 3D screen. However, a deeper depth results in a larger flipping perceived between the viewpoints^17^. Narrowing the projection angle interval of multi-view images is necessary to supress the flipping and smoothen motion parallax. The depth range of the 3D images should be determined such that the flipping is not noticeable. In contrast, when observing the display screen from a position different from the widest effective viewing area, the spatial resolution as a reconstructed optical image affects the spatial resolution characteristics of the 3D images perceived by the viewer. This resolution is mainly determined by the pixel pitch and projection angle interval of the multi-view images and diffusion characteristics of the 3D screen. The resolution limit decreases as the depth distance of the 3D image increases^10^. Consequently, to observe the 3D image with high spatial resolution characteristics and wide depth range at any viewing position, the projection angle interval of multi-view images must be narrowed and the crosstalk amount of reproduced light rays must be suppressed.

Next, Fig. 4b illustrates a 3D image observed from different viewpoints when displaying 3D images of computer graphics (CGs). The occurrence of motion parallax in both the horizontal and vertical directions according to the observation position was confirmed by the change in the positional relation, such as between the heart and blood vessels. The side of the heart could be viewed while moving. Additionally, the front and rear positional relations could be recognised by the viewer in any posture because binocular parallax was also obtained when viewers leaned their heads. The viewing angle was 35.1° in the horizontal direction and 4.7° in the vertical direction, and simultaneous viewing by multiple viewers was possible. A video of the displayed 3D image is provided in the Supplementary Information.

Finally, as shown in Fig. 4c, we displayed a 3D image of real objects and confirmed the image-formation position by setting the diffuser film. When the diffuser film was set 30 mm in front of the 3D screen, the face of the pink mascot character was clearly projected onto the diffuser film. Next, when the diffuser film was moved to 100 mm in front of the 3D screen, the blue rabbit was clearly projected. In this manner, with the proposed method, the formation of the optical images of objects in the depth direction was confirmed. Owing to this feature, 3D images without distortion could be observed independent of the viewing distance and viewer’s posture. A video related to this experimental result is provided in the Supplementary Information.

## Discussion

Unlike conventional display methods based on multi-view projection, Aktina Vision can reconstruct an optical 3D image by reproducing numerous high-density light rays in both horizontal and vertical directions. The 3D screen has a micro-lens array shape, and a slight adjustment of the diffusion angle is easily enabled by changing the refractive indices of the lens and filling materials that constitute the 3D screen. While the diffusion characteristics of the anisotropic diffusing screen used in conventional display methods generally have a Gaussian distribution, the 3D screen used in Aktina Vision has isotropic top-hat diffusion characteristics and narrow diffusion angle of approximately 1.0°. These features can enable the display of a high-resolution full-parallax 3D image while reducing the crosstalk amount between reproduced high-density light rays. In the proposed system, the projection of multi-view images at a narrow angle interval of less than 1.0° is realised by the adoption of a configuration, in which high-density multi-view images displayed by each projector are enlarged and projected onto the 3D screen using the imaging lens and condenser lens. As a result, the reproduction of a large number of light rays can be achieved with a relatively small number of display devices. Although projectors were used as display devices in the prototype system, we intend to develop a thinner display system by using flat-panel display components such as a liquid crystal and organic light-emitting diodes display panels.

In the prototype system, the horizontal projection angle interval of multi-view images is 0.93°, while the FWHM diffusion angle of the 3D screen is 1.12°. Therefore, in a simple calculation, the crosstalk occurs at approximately 20% of the edge of the reproduced light rays. In addition, although the diffusion characteristics of the 3D screen were set to be isotropic, the projection angle interval of multi-view images was different between the horizontal and vertical directions in the prototype display because we used dense multi-view images with an aspect ratio of 16:9. Consequently, the crosstalk amount between reproduced light rays is estimated to be different between their respective directions. This crosstalk is a factor that degrades spatial resolution characteristics of 3D images. These issues can be addressed by changing the curvature of the micro-lens constituting the 3D screen between the horizontal and vertical directions and selecting optimal curable resin materials for the lenses and their fillings. The micro-lens can also have other shapes, such as rectangular and circular. As regards the measurement results of the diffusion characteristics shown in Fig. 2c, the occurrence of diffusion unevenness may be attributed to the diffraction effects. In addition, wide-angle diffusion slightly occurred at the edges of the micro-lens array constituting the 3D screen. To solve these issues, further refinement of the design of the 3D screen, such as achieving optimal spatial randomness in the structure, is considered necessary. In our future work, we intend to improve not only the 3D screen but also the display device and optical system to advance the research and development of this system for practical use.

## Methods

### Compact UHD projector unit

We developed dedicated compact 4K projectors because image information with ultra-high definition and a large number of pixels is needed in Aktina Vision. We realised miniaturisation with a casing size of W 115 mm × H 215 mm × D 680 mm by synthesising three liquid crystals on silicon chips with a size of 0.7 in and full 4K resolution with simple optical prisms and by implementing RGB three-colour light-emitting diode elements as light sources. In addition, by developing the high-resolution projection lens, a high-density image reproduction of 800 ppi with a frame rate of 60 fps was realised. As a result, multi-view images could be displayed without gaps by using multiple projectors placed close together. All projector units display frame-synchronised 4K video replayed by personal computers with frame memories.

### Camera array system

**Figure 5:**
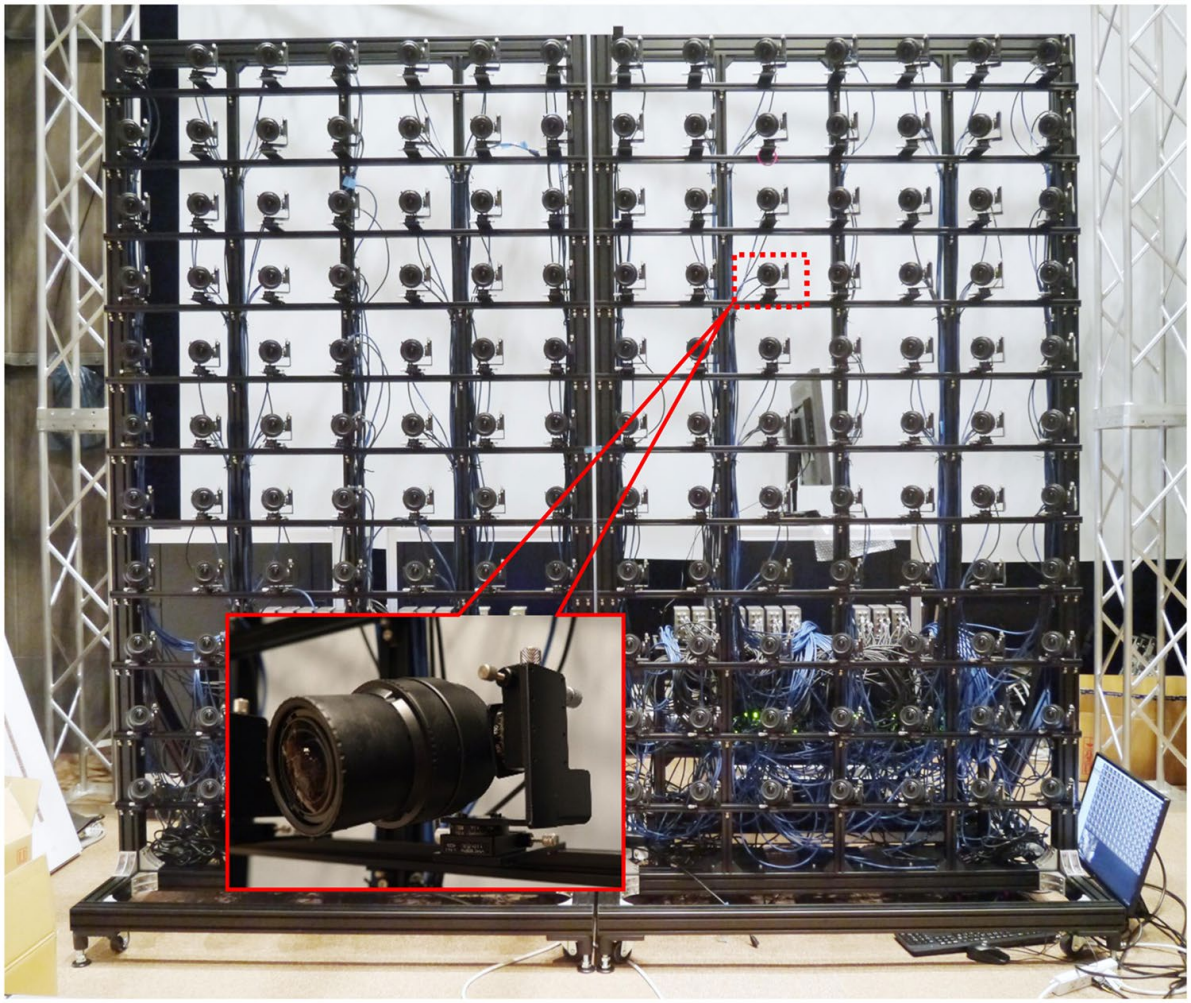
Camera array system. A total of 154 cameras, 14 horizontal and 11 vertical, with a resolution of 1920 × 1080 pixels were used to acquire the light rays of moving real objects. Photo credits: Masanori Kano.

We also developed a camera array system to capture display images for Aktina Vision (Fig. 5). In this system, a total of 154 cameras, 14 horizontal and 11 vertical, with a resolution of 1920 × 1080 pixels were used. The interval between the cameras was set to be approximately 200 mm, the distance to objects was set to approximately 2 m, and the directions of the cameras were centred to the objects to obtain sufficient light rays to display 3D images using the prototype display system. We estimated virtual viewpoint images using the interpolation process of light rays because the number of viewpoints in Aktina Vision is 350, and not all of the required light ray information was captured by 154 cameras.

### Calibration of light field

In practical display systems, errors in light ray reproduction direction occur owing to projection distortions, aberrations in the optical system, and installation errors. Thus, the spatial resolution characteristics of 3D images degrade, and multiple images occur. Therefore, we adjusted the displayed 3D images by geometrically correcting the multi-view images projected by each projector in an offline process. Firstly, a printed reference dot pattern was placed at the same position as the 3D screen. The dot pattern consisted of 9 horizontal dots and 5 vertical dots arrayed in a square pattern. Next, the dot pattern was captured by a calibration camera, and the central position of each dot was detected. Subsequently, the reference dot pattern was replaced with a white screen, and the same shape of the dot pattern was projected by one projector. The reverse-correction was repeated until the projection-position error of each dot was reduced. By applying this process to 350 viewpoints and RGB three-colour separately to correct the projection-position error due to the chromatic dispersion in the optical system, a total of 1,050 correction tables were created. As a result, we could geometrically correct the positional errors in the projected images within approximately ± 250 μm.

## Supplementary information

Video 1

Video 2

Supplementary Information

## Data Availability

The data that support the findings of this study are available from the corresponding author upon reasonable request.
