# Supplementary material for: Aktina Vision: Full-parallax three-dimensional display with 100 million light rays
Source: Sci Rep. 2019 Nov 27;9:17688. doi: 10.1038/s41598-019-54243-6 (PMC6881296; doi:10.1038/s41598-019-54243-6)
Supplement: Supplementary file 3 — Supplementary Information [file 41598_2019_54243_MOESM3_ESM.pdf]

Aktina Vision: Full-parallax three-dimensional display with 100 million light rays

Hayato Watanabe*, Naoto Okaichi, Takuya Omura, Masanori Kano, Hisayuki Sasaki & Masahiro Kawakita

Science & Technology Research Laboratories, NHK (Japan Broadcasting Corporation), Tokyo 157-8510, Japan

*watanabe.h-fe@nhk.or.jp

Supplementary Video 1

A video capture of the displayed 3D image while moving in the horizontal direction, confirming the motion parallax in the wide viewing angle of 35.1°. Video credit: Masanori Kano and Hisayuki Sasaki.

Supplementary Video 2

Video capture of the projected image on the diffuser film while moving the diffuser film away from the display screen. The pink mascot character and blue rabbit are clearly projected on the diffuser film when the position of the diffuser film coincides with the respective imaging positions. This result shows that an optical image of subjects can be reconstructed with the proposed method. Video credit: Masanori Kano and Hisayuki Sasaki.
